# Editorial: Mucosal microbiota immunomodulation of the gut-lung axis

**DOI:** 10.3389/fimmu.2025.1721724

**Published:** 2025-10-24

**Authors:** Yuseok Moon, Susanta Pahari, Shikha Negi, Evelyn Tsantikos

**Affiliations:** ^1^ Laboratory of Food Systems Analytics, Department of Data Science, School of Data Science, Pusan National University, Busan, Republic of Korea; ^2^ Department of Convergence Medical Sciences and Biomedical Research Institute, Pusan National University, Yangsan, Republic of Korea; ^3^ Texas Biomedical Research Institute, San Antonio, TX, United States; ^4^ Cincinnati Children’s Hospital Medical Center, Cincinnati, OH, United States; ^5^ Department of Immunology, School of Translational Medicine, Monash University, Melbourne, VIC, Australia

**Keywords:** gut-lung axis, microbiota, respiratory diseases, mucosal immunity, probiotics, dysbiosis, immune modulation

## 1 Introduction

The concept of the gut-lung axis has emerged as a transformative paradigm in understanding respiratory pathophysiology, fundamentally challenging the traditional organ-centric approach to pulmonary medicine. This bidirectional communication highway connects two mucosal surfaces that collectively harbor the most extensive immune system in the human body, mediated through the mucosa-associated lymphoid tissue (MALT) system (1, 2). The gut microbiota, comprising trillions of microorganisms, functions as a metabolically active organ that profoundly influences systemic immunity through the production of microbial metabolites, including short-chain fatty acids, which regulate immune cell differentiation, maintain epithelial barrier integrity, and modulate inflammatory mediators (3, 4). The establishment of microbial colonization begins at birth and evolves throughout life, with early-life exposures critically shaping respiratory immune competence and disease susceptibility (5). Mounting evidence demonstrates that perturbations in intestinal microbial communities, termed dysbiosis, correlate with diverse respiratory pathologies ranging from chronic obstructive pulmonary disease and asthma to acute respiratory distress syndrome and lung cancer (6). This Research Topic assembles cutting-edge investigations that collectively illuminate the mechanistic underpinnings of gut-lung crosstalk and explore innovative therapeutic strategies targeting this axis for respiratory disease prevention and treatment.

## 2 The microbial landscape: mapping dysbiosis across respiratory conditions

The systematic examination of gut microbiome alterations in chronic lung diseases has yielded compelling evidence of consistent microbial perturbations. Pan et al. provide a comprehensive meta-analysis demonstrating significant gut microbiota imbalances in patients with chronic obstructive pulmonary disease (COPD), characterized by reduced alpha diversity and depleted levels of beneficial genera, including *Bifidobacterium* and *Lactobacillus*, while Bacteroides showed relative enrichment. These findings establish a reproducible microbial signature that transcends individual study populations, suggesting fundamental mechanistic links between intestinal dysbiosis and pulmonary pathology.

The specificity of these microbial alterations extends beyond chronic conditions to acute inflammatory states. Chen et al. identified enrichment of *Prevotella melaninogenica* in the lower respiratory tract of patients experiencing checkpoint inhibitor pneumonitis and radiation pneumonitis, coupled with decreased peripheral blood lymphocyte. This discovery bridges two critical domains: the lung microbiome’s direct contribution to inflammatory lung diseases and its potential as a predictive biomarker for treatment-related complications in oncology. The correlation between bacterial abundance and immune cell populations underscores the bidirectional nature of microbe-immune crosstalk.

## 3 Vulnerability and susceptibility: special populations at risk

Certain populations exhibit heightened vulnerability to gut-lung axis perturbations, revealing the delicate balance required for respiratory health. Dehghani et al. demonstrate that pregnancy exacerbates neutrophilic responses to cigarette smoke exposure, with pregnant mice showing amplified inflammatory cell infiltration in bronchoalveolar lavage fluid and altered gut microbiota composition compared to non-pregnant counterparts. The upregulation of neutrophil chemotaxis or inflammatory genes, including CXCL1, S100A8, and S100A9, alongside reduced cecal short-chain fatty acid (SCFA) levels from an altered microbiota community, indicates how physiological states can modulate gut-lung axis sensitivity to environmental insults.

Immunocompromised individuals face distinct challenges, as elegantly demonstrated by Pulvirenti et al. in their prospective study of patients with Common Variable Immunodeficiency (CVID). The oropharyngeal microbiome in these patients exhibited reduced diversity and expansion of potentially pathogenic genera, including *Haemophilus* and *Streptococcus*, particularly in those with residual IgA deficiency and COPD. Importantly, this microbial dysbiosis correlated with increased risk of acute respiratory infections, suggesting that the oropharyngeal microbiota disturbance as a consequence of mucosal immunoglobulin deficiency, chronic lung injury, or prophylactic antibiotic treatment may contribute to infection susceptibility.

## 4 Mechanistic foundations: unraveling the communication pathways

Understanding how gut microbiota influences pulmonary immunity requires dissecting the molecular mediators of this cross-talk. Yu et al. provide an updated synthesis of mechanisms through which gut microbes modulate pulmonary function, emphasizing roles of immune mediators, metabolites such as short-chain fatty acids, and direct microbial translocation. These pathways collectively enable intestinal microbial communities to exert profound effects on distant lung tissue.

Zhang et al. offer mechanistic insights through their influenza infection model, demonstrating that respiratory viral infection triggers intestinal damage despite viral confinement to the lungs. Their work reveals that altered amino acid metabolism, particularly tryptophan metabolism via indoleamine 2,3-dioxygenase 1 (IDO1), disrupts the Th17/Treg balance with increased *Enterobacteriaceae* and decreased *Lactobacillaceae* in the intestinal tissue. Remarkably, supplementation with tryptophan and *Lactobacillus* ameliorated both pulmonary and intestinal damage, providing proof-of-concept for microbiome-targeted interventions against the distress in the gut-lung axis.

The critical nature of maintaining microbial homeostasis is further emphasized by Zhang et al.'s observation that antibiotic treatment worsened both lung and intestinal pathology following influenza infection, highlighting the protective role of gut commensal microbiota and its metabolic regulation even during acute respiratory infections.

## 5 Clinical implications: from acute lung injury to cancer immunotherapy

The clinical relevance of gut-lung axis perturbations extends across the spectrum of respiratory pathology. Zhang et al. comprehensively review the role of gut microbiota and their metabolites (SCFAs, amino acid metabolites, and bile acid metabolites) in acute respiratory distress syndrome (ARDS), noting that gut microbial alterations influence inflammatory responses, oxidative stress, and multiple cellular pathways, including apoptosis, autophagy, pyroptosis, and ferroptosis, ultimately contributing to the airway distress. Given ARDS mortality rates of 17-39% in general populations and higher rates in COVID-19 patients, understanding microbiome contributions offers urgently needed interorgan communication-based therapeutic opportunities.

Moreover, Sun et al. extend this discussion to multiple respiratory conditions, including pulmonary fibrosis, asthma, and lung cancer, systematically evaluating evidence for microbial therapies. Their analysis raises the crucial question of whether observed associations represent true causal relationships or merely correlations with disease pathology. The need for rigorously designed and large-scale clinical investigations would provide convincing evidence for direct microbial, prebiotic, or metabolite-based postbiotic therapies.

In the oncology realm, Dora et al. provide an interdisciplinary exploration of the gut-lung axis in lung cancer development and immunotherapy responses. Their work emphasizes how microbial dysbiosis and increased gut permeability can precipitate systemic inflammation that influences tumor microenvironments and responses to immune checkpoint inhibitors. This perspective integrates embryological, anatomical, and immunological dimensions of the MALT system, advocating for holistic approaches that consider gut barrier integrity in optimizing cancer treatment outcomes against lung cancer.

## 6 Therapeutic frontiers and emerging issues

Perhaps the most exciting dimension of gut-lung axis research lies in its therapeutic potential. Liu et al. expand our perspective by examining how gut microbial signals influence hematopoietic stem cells (HSCs) and the bone marrow microenvironment, affecting immune cell development at its source. Particularly, gut microbiota is involved in the regulation of HSCs through the bone marrow matrix cells and microbial metabolites. This aligns with emerging evidence that microbial metabolites regulate myelopoiesis and shape the functional properties of circulating immune cells (3, 7). This systems-level perspective necessitates integrative research approaches that simultaneously examine intestinal, pulmonary, and hematopoietic compartments. All evidence suggests that modulation of gut microbiota or its metabolites could fundamentally reshape systemic immunity, with downstream effects on respiratory immune surveillance and airway disease susceptibility.

Although the collective findings presented in this Research Topic advance our understanding of gut-lung axis biology on multiple fronts, there are emerging questions and knowledge gaps requiring additional investigations. First, while observational studies consistently demonstrate associations between gut dysbiosis and respiratory diseases, the directionality and causality of these relationships remain incompletely resolved. Evidence from germ-free animal models demonstrates that the absence of gut microbiota impairs pulmonary immunity and increases susceptibility to respiratory infections, supporting a causal role for the microbiome in respiratory health (8, 9). However, the bidirectional model proposed by Dehghani et al. and the mechanistic insights from Zhang et al. suggest both processes occur simultaneously, creating feedback loops that perpetuate disease states.

Second, the identification of specific microbial signatures, such as *Prevotella melaninogenica* enrichment in checkpoint inhibitor pneumonitis and reduced *Lactobacillus* in COPD (7), raises possibilities for microbiome-based diagnostic and prognostic biomarkers. However, considerable inter-individual variation in baseline microbiome composition, influenced by genetics, diet, geography, medication history, and other environmental factors, complicates the development of universal microbial signatures (10). Future studies must account for these confounding variables through larger, more diverse cohorts and longitudinal sampling strategies that capture temporal dynamics of microbial communities (11, 12).

Third, therapeutic interventions targeting the gut-lung axis show promise but require rigorous validation. Clinical trials examining probiotic interventions in respiratory diseases have yielded mixed results, likely reflecting heterogeneity in strain selection, dosing regimens, and patient populations (13, 14). Translation to human populations necessitates carefully designed randomized controlled trials that consider disease-specific mechanisms, optimal timing of interventions, and potential adverse effects. For example, the observation that antibiotic treatment exacerbates respiratory pathology underscores the importance of antimicrobial stewardship and selective microbiome modulation rather than broad-spectrum elimination.

## 7 Conclusion: an integrated vision

**Figure 1:**
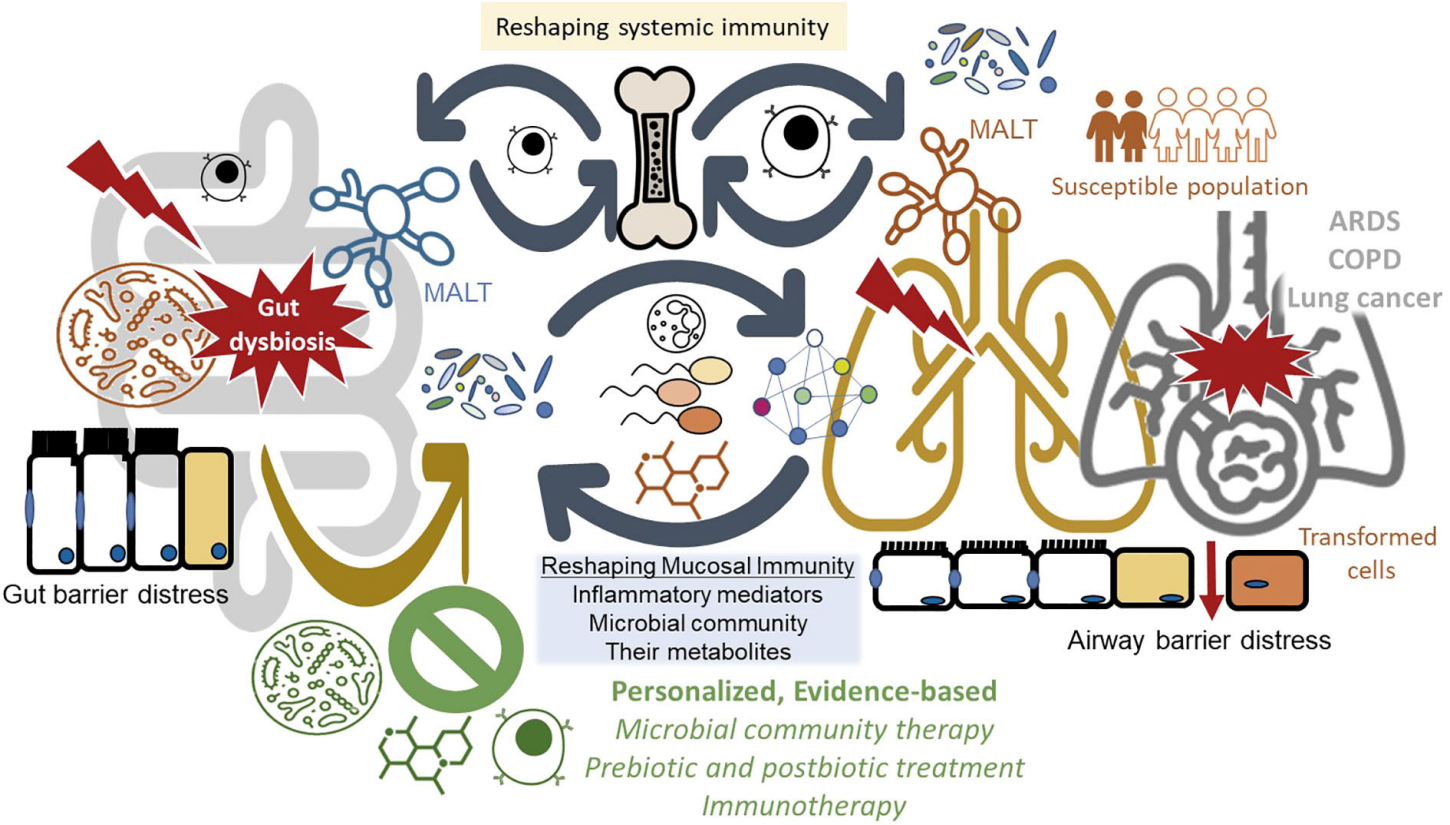
Conceptual framework of gut-lung axis immunomodulation and therapeutic interventions. This schematic illustrates the bidirectional communication pathways and pathological processes within the gut-lung axis, as synthesized from the research topic "Mucosal Microbiota Immunomodulation of the Gut-Lung Axis." The mucosal compartments of both intestinal and respiratory tracts harbor distinct microbial communities that produce bioactive metabolites from dietary substrates and endogenous compounds. Under homeostatic conditions, intact mucosal barriers contain these microbial components within their respective compartments. However, barrier disruption enables translocation of microbes, microbial products, and antigens into the systemic circulation, potentially triggering distant organ pathology. Concurrently, mucosal antigens are captured by antigen-presenting cells within local mucosa-associated lymphoid tissues (MALTs), initiating immune responses that extend beyond the site of antigen encounter, including changes in the bone marrow microenvironment that influence hematopoietic stem cell differentiation and immune cell development. These immune signals reprogram cellular and molecular networks across organ systems, modulating inflammatory pathways and disease outcomes in respiratory conditions, including acute respiratory distress syndrome (ARDS), chronic obstructive pulmonary disease (COPD), and lung cancer. Vulnerability to gut-lung axis dysregulation is heightened in susceptible populations, including pregnant individuals, immunocompromised patients, and those with pre-existing chronic diseases. Therapeutic and preventive strategies targeting this axis encompass microbiota-modulating interventions (probiotics, prebiotics, postbiotics, and fecal microbiota transplantation) and immune-directed therapies, which should be implemented in an evidence-based, safe, and personalized manner to restore mucosal homeostasis and mitigate respiratory disease progression.

The gut-lung axis represents a paradigm of biological integration, where microbial communities serve as both sensors and effectors of health across organ systems. The research compiled in this topic reveals that respiratory diseases cannot be fully understood or optimally treated without considering their intestinal dimensions (**Figure 1**). The convergence of evidence across these ten articles points toward several promising interventional strategies: probiotic supplementation to restore beneficial bacterial populations, prebiotic approaches to nourish protective microbes, postbiotic therapies leveraging microbial metabolites, and fecal microbiota transplantation for comprehensive microbiome reconstitution. However, translating these approaches from experimental models to clinical practice requires careful attention to evidence-based cause-and-effect associations, patient-specific factors, disease contexts, and timing of interventions. In particular, future research must prioritize establishing causality through interventional studies, identifying patient-specific microbial signatures for personalized medicine approaches, and developing risk assessment models that integrate microbiome data with clinical and immunological parameters. The ultimate goal, preventing and treating respiratory diseases through microbiome modulation, moves closer to reality with each insight into this remarkable biological axis.
